# An ecological time series study of lagged associations between pesticide use and cancer incidence in Ukraine

**DOI:** 10.3389/fpubh.2026.1860025

**Published:** 2026-07-01

**Authors:** Mykola Kondratiuk, Anna Antonenko, Sergii Omelchuk, Fedir Melnychuk, Olexandr Kovalchuk, Nazarii Kobyliak, Andrii Borysenko

**Affiliations:** 1 Bogomolets National Medical University, Kyiv, Ukraine; 2 L.I. Medved's Research Center of Preventive Toxicology, Food and Chemical Safety, Kyiv, Ukraine; 3 Taras Shevchenko National University, Kyiv, Ukraine; 4 Medical Laboratory CSD, Kyiv, Ukraine

**Keywords:** breast cancer, cancer incidence, colorectal cancer, ecological study, lag analysis, lung cancer, pesticides

## Abstract

**Introduction:**

Cancer remains a leading cause of mortality and health loss worldwide, and the use of pesticides as potential environmental determinants of oncological risk is being increasingly discussed.

**Materials and methods:**

This ecological time series study assessed the associations between pesticide use in Ukraine (2000–2021) and cancer incidence (2014–2021) using national aggregated data. Cancer-incidence data were obtained from the National Cancer Registry of Ukraine, and pesticide use data were obtained from FAOSTAT and the State Statistics Service of Ukraine. Analyses included total pesticide use and major pesticide groups (fungicides/bactericides, herbicides, insecticides, plant growth regulators, and other pesticides). Given the potential impact of the COVID-19 pandemic on cancer detection and registration during 2020–2021, two analytical scenarios were applied (2014–2021 and 2014–2019). Associations were assessed using Spearman lag correlations (0–14 years) with Benjamini–Hochberg false discovery rate correction. Additional sensitivity analyses included first differences and linear detrending. All analyses were considered hypothesis-generating because of the short time series and ecological study design.

**Results:**

Pesticide use in Ukraine demonstrated wave-like dynamics, with a peak in 2012, a subsequent decline in 2014–2020, and a moderate increase in 2021, whereas herbicides predominated throughout the study period. For total cancer incidence, lag correlations varied substantially by time window and were largely attenuated after detrending and differencing, suggesting sensitivity to shared temporal trends. Short-lag inverse associations were interpreted cautiously because they likely reflected temporal structure rather than immediate biological effects. In site-stratified analyses, partly reproducible positive ecological association patterns within the 5–11-year lag window were observed for female breast cancer, female and male colon cancer, and female trachea/bronchus/lung cancer. The most consistent associations involved herbicides, fungicides/bactericides, insecticides, total pesticide use, and area-based pesticide-use indicators.

**Conclusions:**

These findings are hypothesis-generating and do not support causal inference. Because of the ecological design, ecological fallacy and residual confounding cannot be excluded. Longer time series, refined exposure characterization, and analytical approaches capable of accounting for temporal structure and delayed effects are needed.

## Introduction

Cancer remains among the leading causes of health loss, disability, and mortality worldwide, imposing a substantial burden on health care systems, the economy, and society as a whole. According to global estimates, approximately 19.98 million new cancer cases and 9.74 million deaths from malignant neoplasms were registered in 2022, confirming the exceptional medical and social significance of this problem (1–4). In recent decades, the global cancer burden has increased, driven by population aging, demographic changes, the spread of behavioral risk factors, and the long-term impact of adverse environmental factors (1, 4–6).

Among all malignant neoplasms, particular attention is given to tracheal, bronchial, lung, breast, and colorectal cancer, since these nosology types account for the greatest share of the global cancer incidence burden. According to GLOBOCAN 2022, lung cancer ranked first worldwide in terms of the number of new cases – 2,480,675 (12.4% of all newly diagnosed malignant neoplasms), breast cancer ranked second with 2,296,840 cases (11.5%), and colorectal cancer ranked third with 1,926,425 cases (9.6%) (1–3). Moreover, these three sites are characterized not only by a high prevalence but also by a substantial contribution to mortality and years of life lost, which makes them priority targets for epidemiological, clinical, and preventive research (1, 4).

The selection of these three nosologies for analysis is justified not only by their prevalence but also by the specific features of their etiology. Lung cancer is traditionally associated primarily with tobacco smoking and ambient air pollution; however, in recent years, interest in the role of occupational and environmental chemical exposures in generating additional risk has increased (7–9). The particular importance of breast cancer is attributed to factors related to hormonal regulation, endocrine disturbances, and the long-term accumulation of lipophilic pollutants in body tissues (7, 8, 10). Colorectal cancer, in turn, is among the leading sites for which the role of dietary, household, and environmental chemical exposures is increasingly being discussed, as these may act through oxidative stress, inflammation, genotoxicity, epigenetic changes, and impairment of intestinal barrier function (8, 11). Thus, this triad combines high population significance with the presence of biologically plausible mechanisms of action of environmental determinants.

Pesticides are among the potentially important environmental risk factors for oncopathology. This group of chemical compounds is widely used in agriculture and is capable of entering the human body through occupational, household, dietary, inhalation, and waterborne routes, but the intensity and structure of exposure may substantially depend on the application technology and formulation properties (7, 8, 12–15). According to review studies, chronic pesticide exposure is associated with an increased risk of a number of malignant neoplasms, as well as with neurotoxic, endocrine, immune, and reproductive disturbances (7, 8). Mechanistic studies have summarized several key pathways involved in the potential carcinogenic effect of pesticides, including the induction of oxidative stress, DNA damage, impairment of repair processes, epigenetic changes, endocrine dysregulation, chronic inflammation, and immune dysfunction (8). These mechanisms provide the biological plausibility of possible associations between pesticide exposure and malignant neoplasms. Particular attention has been devoted to endocrine-disrupting, genotoxic, oxidative-stress-related, and chronic inflammatory mechanisms, which may contribute differently depending on cancer site, sex, timing of exposure, and cumulative dose.

Available epidemiological evidence indicates that the relationship between pesticides and cancer risk is heterogeneous; however, for certain sites, sufficient arguments have accumulated to consider it a scientifically meaningful hypothesis. Some of the more consistently reported epidemiological signals have been described for hormone-dependent and digestive tumors, as well as for certain respiratory cancers (7, 8, 16, 17). In the context of breast cancer, pesticides, which are primarily persistent organochlorine compounds, are regarded as potential endocrine disruptors that may affect hormone-sensitive tissues and alter the risk of carcinogenesis (7, 8, 10). An increasing number of studies on colorectal cancer support the hypothesis that certain groups of pesticides contribute to risk formation through chronic low-dose exposure (8, 11). For lung cancer, the evidence base is more heterogeneous because of the substantial influence of tobacco smoking and other confounding factors; however, positive associations have also been described for this site in some occupational and regional studies (7–9). Lung cancer, breast cancer, and colorectal cancer therefore represent biologically and epidemiologically relevant sites for exploratory ecological analysis of possible pesticide-related associations.

For Ukraine, this problem is particularly relevant given the substantial role of the agricultural sector, the widespread use of plant protection products, and the potential unevenness of environmental burden across regions. This issue is particularly relevant for Ukraine, where hygienic studies have already demonstrated that different pesticide application technologies may substantially modify both occupational exposure and potential risks to the population (12, 13, 15). In 2000–2021, according to open statistical sources, substantial fluctuations in pesticide use volumes were observed in Ukraine, while official data from the National Cancer Registry of Ukraine indicate the long-term significance of oncological pathology for public health (18–22). Moreover, 2020–2021 were characterized by the systemic impact of the COVID-19 pandemic on the detection, routing, and registration of cancer cases, which requires a particularly cautious interpretation of time trends (20–22). In this context, assessing the relationships between pesticide use volumes and cancer incidence dynamics in Ukraine is important not only from a scientific but also from a practical point of view, since it may contribute to exploring possible ecological relationships between environmental burden and cancer-incidence dynamics and support risk-oriented approaches in public health.

At the same time, modern environmental-health research increasingly incorporates advanced computational approaches, including machine learning and predictive modeling, to improve the integration of multidimensional exposure indicators, temporal dynamics, and heterogeneous environmental-health datasets. Such approaches may become increasingly important for future pesticide-related epidemiological investigations (23, 24).

From 2000–2021, according to the FAOSTAT (domain “Pesticides Use”) (14), a general trend toward increasing pesticide use levels was observed worldwide, although the distribution of indicators between countries remained sharply asymmetric. The mean value across countries increased from 11,550 thousand tons in 2000 (*n* = 210; 95% CI 5.821–17.290) to 19,030 thousand tons in 2021 (*n* = 211; 95% CI 9.639–28.420), whereas the maximum values in individual countries increased from 430,005 to 719,507 thousand tons. The median also increased from 546.5 thousand tons in 2000 (IQR 70–3.608) to 1,175 thousand tons in 2021 (IQR 121–8.369), reflecting an increase in the “typical” level of use even in the majority of countries, but at the same time, the difference underscores considerable between-country variability and the presence of extreme values (in all years, the minimum was 0). These characteristics indicate that global pesticide use volumes in 2000–2021 increased not only because of individual “leading countries” but also because of a gradual upward shift in the distribution across a wide range of countries, which is important to consider when interpreting ecological and medical-demographic trends (14).

The aim of this ecological time-series study was to assess population-level lagged associations between pesticide use in Ukraine and cancer incidence during 2000–2021 using aggregated national-level indicators, with particular attention to the impact of the COVID-19 period and to priority cancer sites including lung, breast, and colorectal cancer. Given the ecological design and the relatively short national time series, the study was intended as an exploratory and hypothesis-generating analysis rather than an assessment of causal relationships or individual-level exposure effects.

## Materials and methods

### Ethics statement

This study used only publicly available aggregated statistical and registry data and did not involve identifiable human participants; therefore, ethics committee approval and informed consent were not needed.

### Data sources

Data on cancer incidence in the population of Ukraine were obtained from the official bulletins of the National Cancer Registry of Ukraine (indicators per 100 thousand population overall and by age groups). Information on pesticide use (total volumes and by groups of products) was compiled on the basis of open statistical sources, particularly FAOSTAT (pesticides use domain) and data from the State Statistics Service of Ukraine (14, 25). For the analysis, indicators for the main pesticide groups (fungicides/bactericides, herbicides, insecticides, growth regulators, and other pesticides), an integral indicator of total use, as well as indicators of application intensity (kg/ha) from FAOSTAT and a calculated indicator (based on data on the area to which pesticides were applied, kg/ha (State Statistics Service)) were used. Thus, analyses were performed both for total pesticide-use indicators and separately for major pesticide groups.

### Design and time windows

This study was conducted as an ecological analysis of time series during the prewar period. Given the possible systemic impact of the COVID-19 pandemic on the availability of diagnostics and the completeness of case registration from 2020–2021, two analysis scenarios were applied: (1) a fixed “oncology” window from 2014–2021 and (2) a fixed “oncology” window from 2014–2019 (without the pandemic years). These two analytical scenarios were considered complementary sensitivity-analysis approaches rather than directly interchangeable epidemiological estimates. To assess delayed associations, a lag analysis was performed with a shift of the pesticide block relative to the oncological block by 0–14 years with a step of 1 year; in this case, the “oncological” block remained fixed, while the “pesticide” block was shifted in time according to the specified lag. The maximum lag period of 14 years was determined by the availability of national cancer-incidence data beginning in 2014 and pesticide-use statistics beginning in 2000, which represented the maximum technically feasible overlap between the exposure and oncological time windows. All analyses were hypothesis-generating because of the short length of the available time series.

### Statistical analysis

Given the short time series and the potential nonstationarity of the indicators, nonparametric Spearman rank correlation (ρ) was chosen as the main method for assessing associations. For each lag, pairwise correlations were calculated between the indicators of the pesticide block and the indicators of the oncological block (overall indicator and age groups). Multiple testing within each lag was adjusted by the Benjamini–Hochberg false discovery rate control method (q(BH)); the level of statistical significance was set at α = 0.05 with the display of significance gradations (^*^ ≤ 0.05, ^**^ ≤ 0.01, ^***^ ≤ 0.001).

To assess the sensitivity of the results to trends and structural shifts, approaches to working with short series were additionally considered: analysis of increments (Δ-series) and analysis after linear detrending by year (models of the indicator ~ “Year” type using residuals/detrended values). First-difference analysis was performed by calculating year-to-year changes in pesticide-use and cancer-incidence indicators prior to correlation analysis. Linear detrending was performed by fitting regression models of the indicator ~ “Year” type and subsequently using residual values for lag-correlation analysis.

Formal stationarity and autocorrelation diagnostics were not used as primary analytical procedures because of the short effective duration of the lag-specific time series, particularly within the 2014–2019 analytical scenario. Therefore, detrending, first-difference analysis, and comparison of alternative analytical windows were applied as sensitivity approaches to evaluate the potential influence of shared temporal trends and structural shifts.

Alternative time-series approaches, including distributed lag models, ARIMA-type models, and generalized additive models, were considered conceptually; however, they were not applied because the available national time series were insufficiently long for stable parameter estimation and reliable model diagnostics. Descriptive statistics are presented as medians and ranges/interquartile intervals or mean values with variability indicators depending on the nature of the distribution and the visualization task.

### Cartographic analysis and visualization

For the spatial interpretation of trends, thematic maps by regions of Ukraine were constructed: maps of cancer incidence dynamics (2014–2021), as well as graphs of time trends (national level). Visualizations were used to identify general trends, regional heterogeneity, and potential “anomalous” years.

### Software

Primary data organization and part of the calculations were performed in Microsoft^®^ Excel^®^ for Microsoft 365 and MedStat v5.2. Extended statistical analysis (lag correlations, BH-FDR) and the construction of graphs/maps were performed in the Jupyter Notebook environment (Python: pandas, scipy, statsmodels, geopandas/matplotlib).

## Results

**Figure 1:**
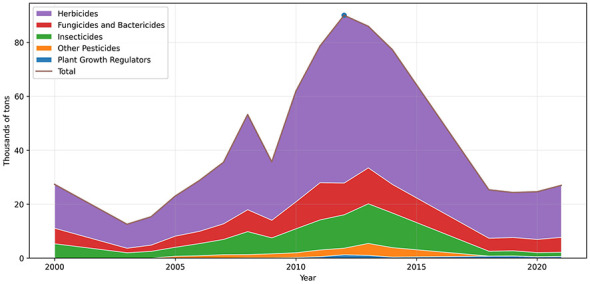
Pesticide use in Ukraine by type (2000–2021).

The presented data for Ukraine (FAOSTAT, Pesticides Use domain) (14) demonstrate pronounced wave-like dynamics of pesticide use from 2000–2021 (Figure 1).

From 2000–2003, the total volume of use decreased from 27,344 tons to 12,558 tons, after which in 2004–2013, a substantial increase was observed, with a peak in 2012 – 90,050 tons (approximately 3.3 times higher than that in 2000). From 2014–2020, a steady decrease to 24,325–24,622 tons was subsequently observed, and in 2021, a moderate increase to 26,974 tons occurred. In terms of use structure in all years, herbicides dominated (for example, in 2012, 62,187 tons, approximately 69% of the total volume), whereas the shares of insecticides and fungicides/bactericides were smaller (in 2012, 12,406 tons and 11,754 tons, respectively). The FAOSTAT use intensity indicator (kg/ha) increased from 0.4–0.8 kg/ha at the beginning of the period to a maximum of 2.7 kg/ha in 2012, after which it decreased to 0.7–0.8 kg/ha in 2019–2021. A similar trend is reflected by the calculated indicator based on data from the national databank (kg/ha), with a maximum of 5.78 kg/ha in 2012 and a decrease to ≈1.55–1.69 kg/ha in 2019–2021 (25).

**Figure 2:**
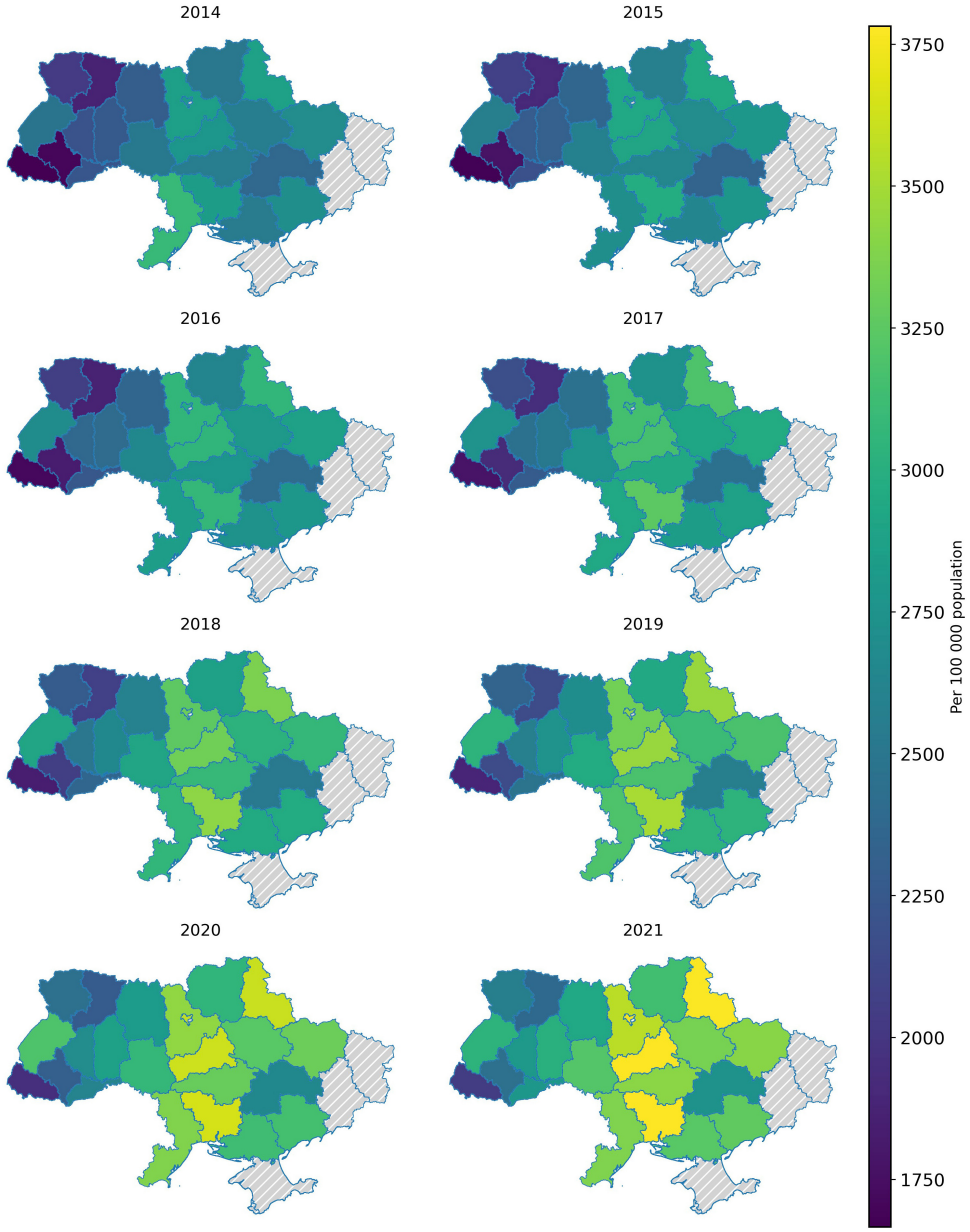
Dynamics of cancer incidence in Ukraine (by region).

In 2014–2021, in most regions of Ukraine, predominantly positive dynamics of cancer incidence were observed, on average, approximately +2–6% per year depending on the region, with the most pronounced increase in 2016–2018; in some regions, the increase reached 5–7% per year. Cartographic analysis confirmed that the increase had not only a temporal but also a spatial characteristic: by 2019–2020, higher levels of the indicator had formed in most regions, with the subsequent preservation of generally high values in 2021 (Figure 2). Deviations from this tendency were isolated: in the Odesa region, a decrease was recorded in 2014–2015 (3090.2 → 2722.6 per 100 thousand; −11.9%) and again in 2020–2021 (3378.0 → 3368.7; −0.3%), in the Rivne region—in 2015–2016 (1910.1 → 1866.9; −2.3%), and in the Lviv region—in 2020–2021 (3187.0 → 3037.2; −4.7%). At the national level, in 2014–2020, the increase remained positive and was +1.4% in 2014–2015, +4.5% in 2017–2018, and +4.4% in 2019–2020, whereas in 2020–2021, a decrease of 12.4% was registered, which probably reflects the impact of the COVID-19 pandemic on case detection and registration rather than a real reduction in the cancer burden.

Cumulatively, from 2014–2021, the greatest cumulative increase was recorded in the Ivano-Frankivsk region (43.6%), Cherkasy region (36.3%), Kyiv city (33.7%), Kirovohrad region (33.6%), Khmelnytskyi region (33.4%), Mykolaiv region (33.1%), and Sumy region (31.0%). The smallest cumulative increase was noted in the Odesa region (9.0%) and the Dnipropetrovsk region (15.5%), which underscores the substantial interregional variability of the trends. Overall, in Ukraine, the cumulative indicator for 2014–2021 was 7.0%, which is a consequence of the positive dynamics up to 2020 and its sharp “collapse” in 2020–2021 against the background of pandemic restrictions.

**Figure 3:**
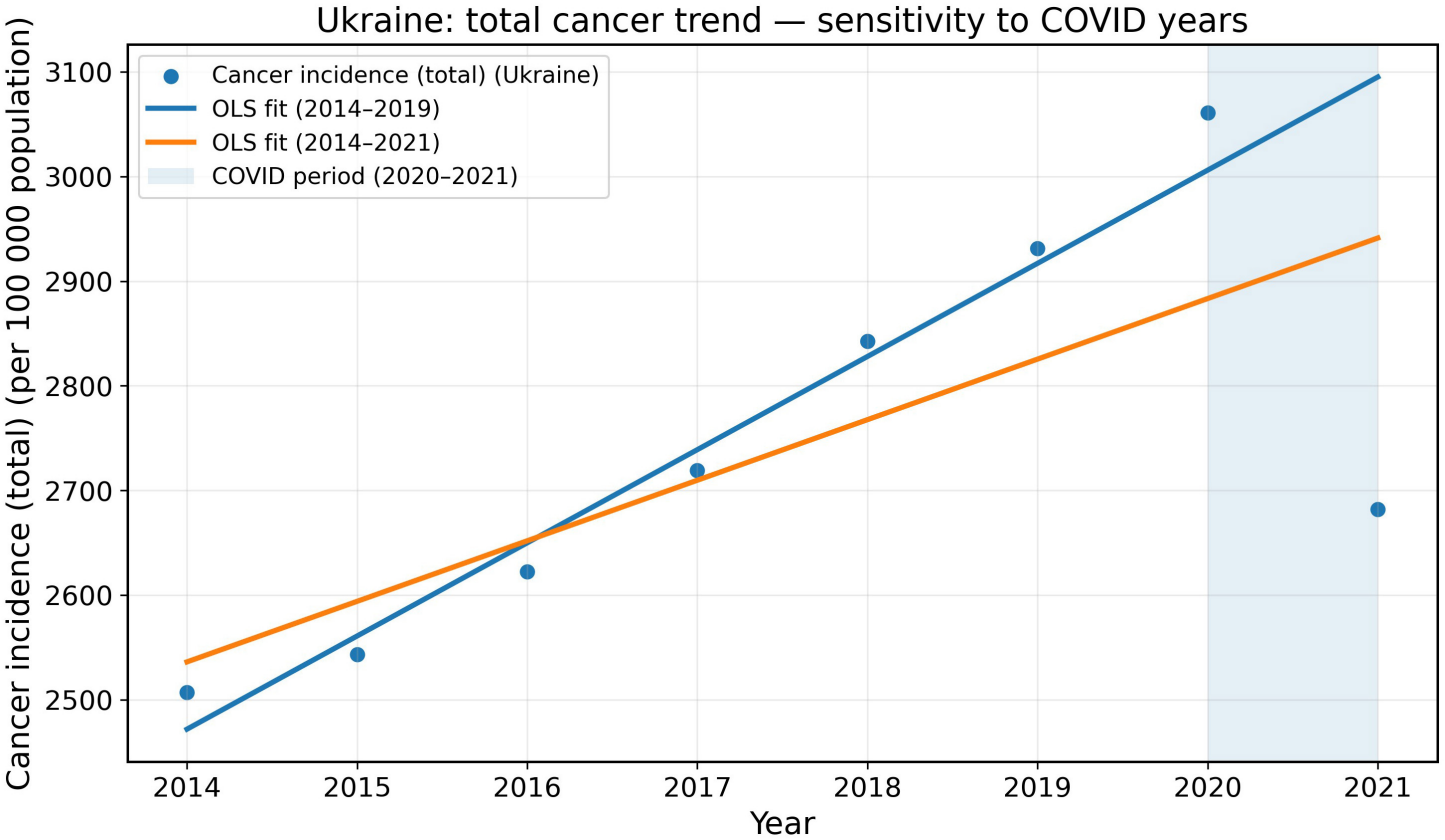
Total cancer incidence trend in Ukraine (2014–2021).

This assumption is confirmed by trend analysis: in 2014–2019, a steady trend toward an increase in the overall incidence indicator was observed. Linear regression (2014–2019): “Total cancer incidence”/“Year” demonstrated a strong direct association (*r* = 0.989; *r*^2^ = 0.978; *p* < 0.001; *n* = 6); the inclusion of 2020–2021 substantially reduced the trend estimate (*r* = 0.735; *r*^2^ = 0.540; *p* = 0.038; *n* = 8) and worsened the behavior of the residuals, which is consistent with a possible structural shift of the indicator during the pandemic years. A diagnostic assessment of the influence of observations revealed that the year 2021 had the greatest Cook's distance; that is, it was the most influential point for the model parameters (Figure 3).

Considering the short and nonstationary national time series (2014–2021), a Spearman lag correlation analysis was performed between total cancer incidence (per 100 thousand population) and the pesticide use indicator (the number of applied pesticides per unit area according to the State Statistics Service and FAOSTAT, also taking into account volumes by class). Because of the ecological design and aggregated national-level structure of the dataset, these associations should be interpreted as population-level temporal relationships rather than as evidence of individual-level exposure effects.

For total cancer incidence, the lag structure of the associations with pesticide use indicators clearly depended on the choice of time scenario. In the 2014–2021 scenario, at lags of approximately 12–5 years, positive correlations predominated for most pesticide indicators, with the most pronounced cluster occurring at lags of 7–6 years. After Benjamini–Hochberg correction, statistical significance was retained at a lag of 7 years for FAOSTAT kg/ha (ρ = 0.91), pesticides (total) (ρ = 0.93), and plant growth regulators (ρ = 0.95) and at a lag of 6 years for insecticides (ρ = 0.95). In contrast, at lag 0 years, which corresponded to the synchronous comparison 2014–2021/2014–2021, the direction of associations changed to the opposite, and strong negative associations dominated: for state statistics kg/ha, herbicides, other pesticides, and pesticides (total), as well as for FAOSTAT kg/ha, the coefficients reached ρ = −0.95, whereas for fungicides and bactericides, ρ = −0.91; these associations also remained significant after BH-FDR correction. Thus, in the full time window, the profile changed from predominantly positive associations at medium lags to strong negative associations at lag 0, which indicates the high sensitivity of the results to the mutual alignment of the exposure and oncological windows. Detailed lag-correlation matrices for the overall cancer incidence indicator are presented in Supplementary Table S1.

After exclusion of the pandemic years, the picture became much sharper. In the 2014–2019 scenario, the absolute values of the coefficients increased, and the number of BH-FDR-confirmed results substantially increased. The most pronounced positive block was concentrated at lags of 11–6 years, which corresponded to exposure windows of approximately 2003–2013. At a lag of 11 years, for State Statistics kg/ha, FAOSTAT kg/ha, Fungicides and Bactericides, Herbicides, Insecticides, and Pesticides (total), the coefficients reached ρ = 1.00, whereas for Other Pesticides, they remained very high (ρ = 0.93). At lags of 10–7 years, high positive correlations were stably preserved: for Fungicides and Bactericides, Insecticides, Other Pesticides, and Pesticides (total), values of ρ = 0.94 were repeatedly observed; for FAOSTAT kg/ha, ρ = approximately 0.90; and for Plant Growth Regulators, the peak values of ρ = 0.99–1.00 occurred at lags of 9–7 years. At a lag of 6 years, after BH-FDR correction, positive associations were also retained for State Statistics kg/ha and FAOSTAT kg/ha (ρ = 0.89), Herbicides (ρ = 0.89), Insecticides (ρ = 0.94), Other Pesticides (ρ = 0.94), Pesticides (total) (ρ = 0.89), and Plant Growth Regulators (ρ = 0.94). Thus, in the pre-COVID-19 scenario, the most stable positive lag window for total cancer incidence fell approximately 11–6 years, with the preservation of elevated coefficients up to a lag of 5 years (Supplementary Table S1).

Moreover, in the 2014–2019 scenario, a distinct inverse block also formed at the smallest lags. At lags of 2–0 years, which corresponded to the latest shifted windows of pesticide exposure, strong negative correlations dominated for most indicators. In particular, for State Statistics kg/ha, Herbicides, and Pesticides (total), the values reached ρ = −1.00 at lags of 2–0 years; for FAOSTAT kg/ha, ρ = −1.00 at lags of 2–0 years; for Fungicides and Bactericides, ρ = −0.94–1.00; for Insecticides, ρ = −1.00 at lag of 1 year; and for Other Pesticides, ρ = −1.00 at lags of 1–0 years. A substantial portion of these negative associations also retained significance after BH-FDR correction. Thus, for total cancer incidence, the lag structure was not monotonic but included a pronounced zone of positive correlations at medium lags and a strong inverse block at small lags; after exclusion of the pandemic years, these associations became substantially stronger and more numerous (Supplementary Table S1).

Such a distribution of the results is consistent with further interpretation: the strongest synchronous or short-lag negative correlations probably reflect primarily the trend structure of short ecological series, whereas the positive block at medium lags is more meaningful from the etiological point of view. However, given the ecological design, short time series, multiple lag comparisons, and absence of direct control for important confounding variables, even BH-FDR-confirmed associations should be regarded only as hypothesis-generating rather than as evidence of a causal relationship (Supplementary Table S1).

Taking into account the short and nonstationary national time series (2014–2021), a Spearman lag-correlation analysis was performed between total cancer incidence (per 100 thousand population) and the pesticide use indicator according to the State Statistics Service (kg/ha) for lags of 0–14 years. In the original series levels, a pronounced lag-dependent change in the direction and strength of associations was revealed. For small lags, strong negative associations were observed: at lag 0 years ρ = −0.95 (*p* < 0.001; 95% bootstrap CI from −1.00–0.62), at lag 1 year ρ = −0.86 (*p* = 0.007; 95% CI from −1.00–0.32), and at lag 2 years ρ = −0.76 (*p* = 0.028; 95% CI from −1.00–0.06). In contrast, in the lag range of 6–12 years, positive associations predominated, with a maximum at a lag of 7 years (ρ = 0.88; *p* = 0.004; 95% CI 0.39–1.00). High positive coefficients were also observed at a lag of 6 years (ρ = 0.81; *p* = 0.015; 95% CI 0.16–1.00), 8 years (ρ = 0.74; *p* = 0.037; 95% CI 0.04–1.00), and 12 years (ρ = 0.86; *p* = 0.007; 95% CI 0.34–1.00). After correction for multiple comparisons by the FDR method, lags of 0, 1, 6, 7, and 12 years remained formally statistically significant (*q* = 0.004; 0.025; 0.045; 0.025 and 0.025, respectively), indicating the presence of a pronounced but unstable lag-dependent structure of associations at the original levels.

After linear detrending by year, most lags did not demonstrate stable associations: for the predominant part of lags, the 95% bootstrap confidence intervals crossed zero, and the *p*-values exceeded 0.05. Moreover, at lag 1 year, a strong negative association remained (ρ = −0.86; *p* = 0.007; 95% CI from −1.00–0.31), and at lag 12 years, a strong positive association remained (ρ = 0.83; *p* = 0.010; 95% CI 0.26–1.00). However, after FDR correction, for both lags, the *q*-values were 0.076; that is, they did not reach the formal threshold of statistical significance. For lags 6–8 years after detrending, moderate positive coefficients were observed (ρ = 0.52; 0.52; 0.43, respectively), but their confidence intervals were wide and included zero (from −0.34–0.97; from −0.29–1.00; and from −0.47–1.00, respectively).

**Figure 4:**
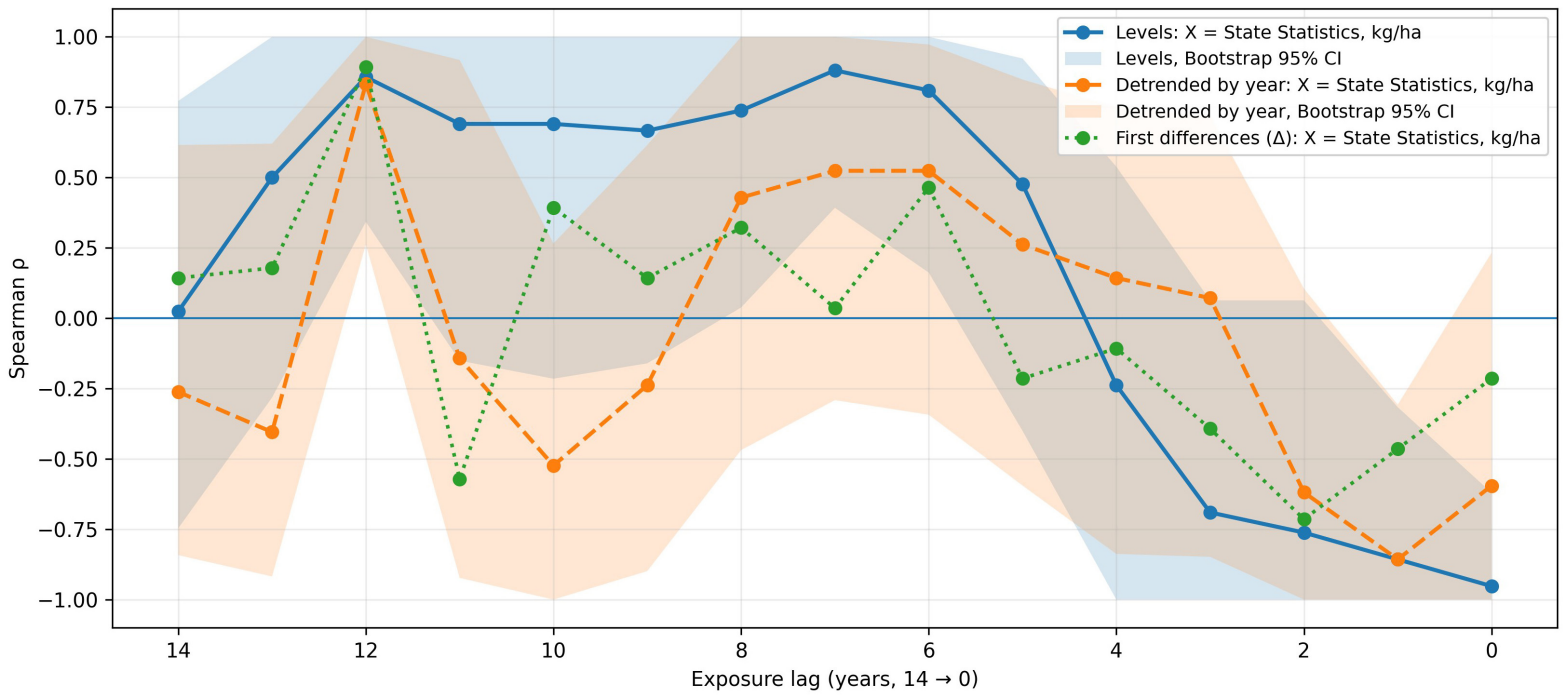
Ukraine: lagged correlations between cancer incidence (2014–2021) and pesticide application rate (state statistics, kg/ha) in levels, detrended series, and first differences.

The analysis of first differences (Δ) also did not reveal a stable consistent signal: most coefficients were unstable in sign and magnitude, and the *p*-values did not reach statistical significance. The largest positive coefficient was observed at a lag of 12 years (ρ = 0.89; *p* = 0.007), but after correction for multiple comparisons, this result also lost formal statistical significance (*q* = 0.102). Overall, the combination of pronounced associations in the original levels, their substantial weakening after detrending, and the absence of a stable signal in the analysis of first differences indicate that a significant part of the revealed lag correlations is probably caused by common temporal trends rather than by an autonomous causal relationship. These findings additionally suggest that the observed associations were highly sensitive to the temporal structure of the short national series and that residual false-positive findings cannot be completely excluded despite BH-FDR correction. Given the very short time sample (*n* = 8 for each lag) and the multiplicity of the tested lags, these results should be regarded as preliminary and hypothesis-generating; they require confirmation on longer time series with control of potential confounding factors (Figure 4).

**Figure 5:**
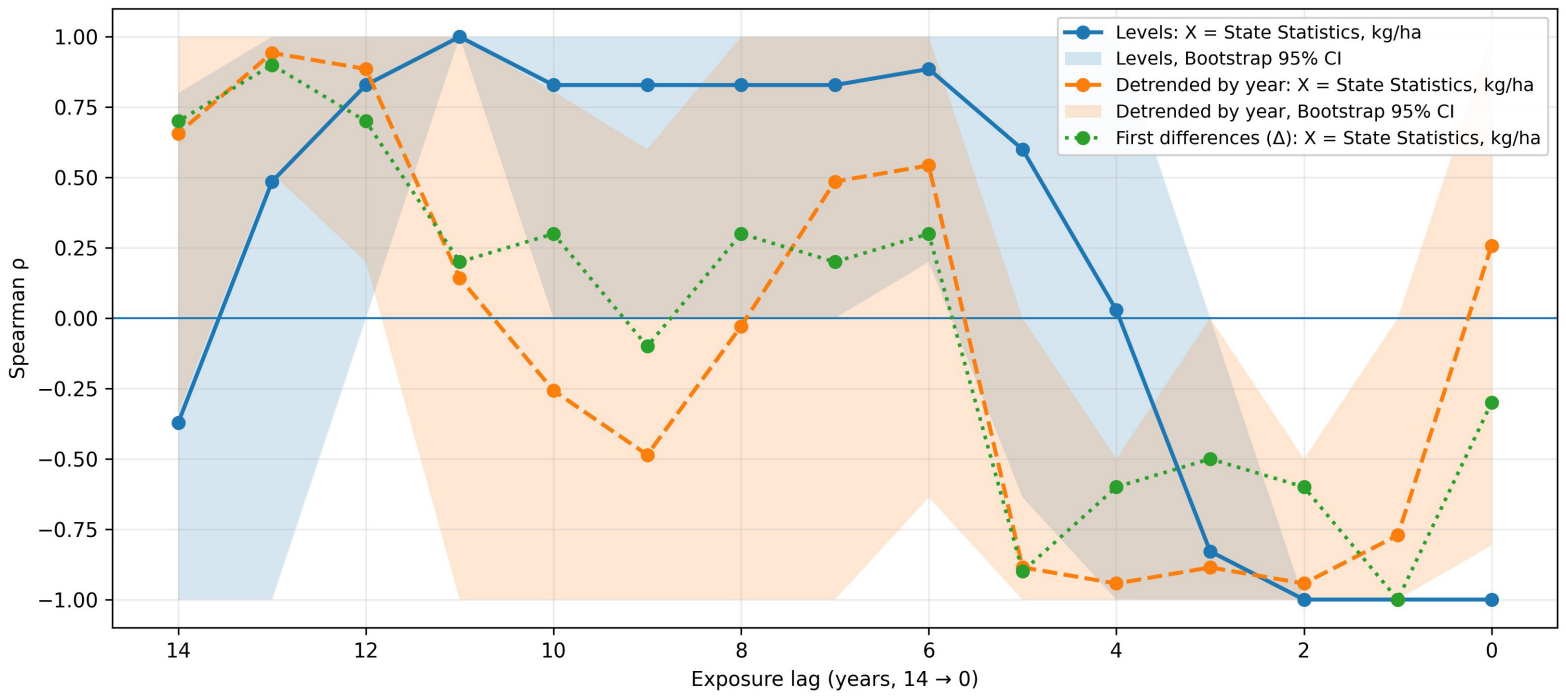
Ukraine: lagged correlations between cancer incidence (2014–2019) and pesticide application rate (state statistics, kg/ha) in levels, detrended series, and first differences.

Given the short pre-COVID-19 national series (2014–2019), a Spearman lag correlation analysis was performed between total cancer incidence (per 100 thousand population) and the pesticide use indicator according to the State Statistics Service (kg/ha) for lags of 0–14 years. In the original series levels, a pronounced lag-dependent change in the sign and strength of associations was revealed. For small lags, very strong negative associations were observed: at lags 0, 1, and 2 years, the coefficient was ρ = −1.00; for lags 0 and 1, *p* < 0.001; for lag 2, *p* = 0.005; and the 95% bootstrap CIs for all three lags were shifted toward negative values (from −1.00–1.00; from −1.00–1.00; and from −1.00–0.50, respectively). At lag 3 years, a strong negative association also remained (ρ = −0.89; *p* = 0.019; 95% CI from −1.00–0.00), and at lag 4 years, the association was similar in magnitude (ρ = −0.94; *p* = 0.005; 95% CI from −1.00–0.50). Starting from lag 6 years, the direction of the association changed to positive: at lags 6 and 12 years, strong positive correlations were detected (ρ = 0.89; *p* = 0.019; 95% CI 0.20–1.00 and 0.20–1.00, respectively), and at lag 13 years, an even stronger positive association was detected (ρ = 0.94; *p* = 0.005; 95% CI 0.52–1.00). The maximum positive association was observed at a lag of 11 years (ρ = 1.00; *p* < 0.001; 95% CI 1.00–1.00). After correction for multiple comparisons by FDR, lags of 0, 1, 2, 3, 4, 11, 12, and 13 years remained formally significant (*q* = < 0.001; < 0.001; 0.024; 0.047; 0.024; < 0.001; 0.047 and 0.024, respectively) (Figure 5).

After linear detrending by year, the overall picture changed substantially. For most lags, the 95% bootstrap confidence intervals remained wide, and the results were unstable in sign. Moreover, strong negative associations remained at lags 2 and 4 years (ρ = −0.94; *p* = 0.005; 95% CI from −1.00–0.50 for both), as well as at lags 3 and 5 years (ρ = −0.89; *p* = 0.019; 95% CI from −1.00 to 0.00 for both). After FDR correction, lags 2, 3, 4, 5, 12, and 13 years remained formally significant (*q* = 0.024; 0.047; 0.024; 0.047; 0.047 and 0.024, respectively). Moreover, positive associations after detrending were observed predominantly at longer lags, particularly at a lag of 12 years (ρ = 0.89; *p* = 0.019; 95% CI 0.20–1.00) and a lag of 13 years (ρ = 0.94; *p* = 0.005; 95% CI 0.52–1.00), whereas at a lag of 14 years, the association was moderately positive but statistically nonsignificant (ρ = 0.66; *p* = 0.156; 95% CI from −0.33–1.00).

Analysis of the first difference (Δ) did not reveal a convincingly stable signal. Formally, the strongest negative association was observed at lag 1 year (ρ = −1.00; *p* < 0.001; *q* < 0.001), but given the very small number of observations for the analysis of increments (*n* = 5), these results should be interpreted with extreme caution. For most other lags, the coefficients were unstable in sign and magnitude and did not reach statistical significance after FDR correction. In general, the combination of extreme coefficients in levels, wide confidence intervals, and a sharp change in the structure of associations after detrending indicates that for the pre-COVID period, lag correlations are very sensitive to the trend component and the small sample size. Given that for levels and detrended series *n* = 6 and for first differences *n* = 5, the results should be regarded as purely preliminary and hypothesis-generating.

In the nosologically stratified analysis, lag associations between pesticide use indicators and cancer incidence varied by sex, cancer site, and duration of the time shift between the exposure window and the cancer incidence window. In contrast to the overall cancer incidence indicator, where the lag structure reflected mainly unstable temporal patterns of the short national series, more distinct and partly reproducible correlation signals were identified for selected priority cancer sites. The two most contrasting intervals were short lags of 0–1 year, where inverse associations predominated, and the 5–11-year lag window, where positive associations partly persisted after Benjamini–Hochberg correction. A summary of BH-FDR-confirmed positive associations is presented in Table 1, whereas the complete lag-correlation matrices are provided in Supplementary Tables S2–S4.

**Table 1:** Summary of BH-FDR-confirmed positive lag associations between pesticide use indicators and selected cancer sites in Ukraine (2014–2019 scenario).

Cancer site	Sex	Lag window (years)	Main pesticide indicators with BH-FDR-confirmed positive associations	Direction of association
Breast cancer	Female	7–10	State Statistics kg/ha, FAOSTAT kg/ha, herbicides, fungicides/bactericides, insecticides, other pesticides, pesticides (total)	Positive
Colon cancer	Female	7–10	FAOSTAT kg/ha, insecticides, other pesticides, pesticides (total)	Positive
Colon cancer	Male	7–10	State Statistics kg/ha, FAOSTAT kg/ha, herbicides, fungicides/bactericides, insecticides, plant growth regulators, other pesticides, pesticides (total)	Positive
Trachea, bronchus, and lung cancer	Female	5–10	FAOSTAT kg/ha, fungicides/bactericides, insecticides, other pesticides, plant growth regulators, pesticides (total)	Positive

The table summarizes only BH-FDR-confirmed positive associations considered biologically interpretable within the delayed 5–11-year exposure window. Full lag-correlation matrices are provided in Supplementary Tables S1–S4. No reproducible block of BH-FDR-confirmed positive associations was observed for male breast cancer or male trachea, bronchus, and lung cancer.

At lags of 0–1 year, strong inverse correlations predominated in most female models and in male colon cancer. This pattern was most evident for breast cancer, colon cancer, and trachea, bronchus, and lung cancer in women, as well as for colon cancer in men. Given the long latent and preclinical course of these malignancies, such short-lag inverse associations are unlikely to reflect an immediate biological effect of current exposure. More plausibly, they reflect the nonstationarity of the time series, common macrotrends, peculiarities of case detection and registration, and statistical mismatch between the current pesticide burden and the already established cancer burden.

In contrast, the most meaningful positive signal was observed in the 5–11-year lag window. For female breast cancer, BH-FDR-confirmed positive associations were concentrated mainly at lags of 7–10 years and included State Statistics kg/ha, FAOSTAT kg/ha, herbicides, fungicides/bactericides, insecticides, other pesticides, and pesticides (total). For female colon cancer, the most consistent positive associations in the same lag window involved FAOSTAT kg/ha, insecticides, other pesticides, and pesticides (total). For female trachea, bronchus, and lung cancer, BH-FDR-confirmed positive associations covered lags of 5–10 years and were observed for FAOSTAT kg/ha, fungicides/bactericides, insecticides, other pesticides, plant growth regulators, and pesticides (total). For male patients with colon cancer, the clearest positive signal was identified within a lag of 7–10 years and included State Statistics kg/ha, FAOSTAT kg/ha, herbicides, fungicides/bactericides, insecticides, plant growth regulators, other pesticides, and pesticides (total). No stable BH-FDR-confirmed positive association pattern was identified for male breast cancer or for male trachea, bronchus, and lung cancer.

Taken together, these findings indicate that any potentially meaningful ecological signal is not universal across cancer sites but rather site- and sex specific. The concentration of the most reproducible positive associations within the 5–11-year lag window makes this interval the most relevant for cautious etiological interpretation; however, given the ecological design, short time series, and multiple testing, these results should be regarded as preliminary and hypothesis-generating rather than causal.

## Discussion

In short national time series, the associations between pesticide use indicators and cancer incidence in Ukraine are heterogeneous in both direction and magnitude and are highly sensitive to the choice of time window, cancer site, sex, and method of time series processing. This pattern is expected for short ecological time series with pronounced temporal structure, in which shared trends, structural shifts, and multiple lag comparisons may generate unstable or potentially spurious correlations. Moreover, despite the overall instability, partly reproducible lag-related ecological patterns were identified for selected cancer sites, which warrants cautious interpretation.

For total cancer incidence, lag analysis revealed a marked shift in the direction of associations: strong inverse correlations predominated at short lags, whereas positive correlations were more common in the 6–12-year range. However, these findings were substantially attenuated after linear detrending and first-difference analysis, and most confidence intervals crossed zero. This finding suggests that a substantial part of the signal observed for the aggregated cancer-incidence indicator may reflect shared temporal trends rather than an autonomous exposure-outcome relationship. A similar pattern was observed in the pre-COVID window of 2014–2019, where very high coefficients were detected in the original series but remained highly sensitive to the trend component and the small sample size. Therefore, for the overall national cancer incidence indicator, the observed associations should be interpreted primarily as hypothesis-generating.

Particular caution is needed when interpreting the strong inverse associations observed at lags of 0–1 year, which recurred in most female models and in male colon cancer. From a biological perspective, such short-lag associations are unlikely to reflect a direct carcinogenic effect of current pesticide exposure, given the long latent and preclinical course of breast, colorectal, and trachea/bronchus/lung cancers. A more plausible explanation is that these inverse relationships reflect the nonstationarity of the time series, common macrotrends, variation in case detection and registration, and statistical mismatch between the current pesticide burden and the already formed cancer burden. In this context, the short-lag inverse block is better interpreted as a marker of temporal structure than as evidence of an immediate biological effect.

In contrast, the 5–11-year lag window yielded the most consistent and biologically plausible signal. In this interval, positive associations partly persisted after BH-FDR correction for female breast cancer, female and male colon cancer, and female trachea/bronchus/lung cancer. Among the partly reproducible ecological patterns, the most consistent positive associations were observed for total pesticide use, herbicides, fungicides/bactericides, insecticides, and both area-based indicators (FAOSTAT kg/ha and State Statistics kg/ha). In men with trachea/bronchus/lung cancer, inverse rather than positive coefficients predominated in the same lag window, whereas no reproducible block of BH-FDR-confirmed positive associations was detected for male breast cancer. Taken together, these findings suggest that any potentially meaningful ecological signal is site- and sex-specific rather than universal.

From a medical and biological perspective, a delayed effect of pesticide exposure is plausible, since carcinogenesis in many malignancies occurs over years or decades. Experimental and mechanistic studies have indicated that pesticides may contribute to procarcinogenic processes through oxidative stress, DNA damage, impaired repair, endocrine dysregulation, chronic inflammation, immune dysfunction, and altered control of apoptosis and proliferation (8). Within this framework, the concentration of the most reproducible positive associations in the 5–11-year window may cautiously align with the latent course of some malignancies. Such an interpretation appears most plausible for breast and colorectal cancer and may also be considered for female trachea/bronchus/lung cancer, although the latter requires particular caution because of the likely contribution of smoking, ambient air pollution, and other major risk factors.

These findings are broadly consistent with the epidemiological literature, which suggests that pesticide-related cancer risk is heterogeneous and depends on the specific active substances, mixtures, routes of entry, and level of personal protection (7, 17). However, most epidemiological evidence comes from occupational cohorts or case–control studies with more specific exposure contexts than those available in ecological national datasets. In our study, country-level pesticide use indicators were used and therefore did not reflect individual dose, route-specific exposure, mixture composition, regional heterogeneity, occupational exposure, or protective practices. Accordingly, direct extrapolation from occupational findings to the general population is limited, and even BH-FDR-confirmed associations should not be interpreted as evidence of causality but rather as statistically reproducible ecological signals requiring further verification.

The sensitivity of the results to the inclusion of 2020–2021 is consistent with probable underregistration of cancer cases during the COVID-19 period and/or reduced access to diagnostics and screening. This represents an important source of systematic distortion in national incidence indicators. Therefore, the 2014–2021 and 2014–2019 analyses should be interpreted as complementary sensitivity-analysis scenarios rather than directly interchangeable epidemiological estimates. The use of two analytical scenarios, together with influence diagnostics and sensitivity analyses, strengthens the conclusion that part of the short-lag structure was shaped by temporal disruptions in the series rather than by biologically meaningful exposure effects.

Regional mapping also revealed substantial spatial heterogeneity in cancer incidence dynamics across Ukraine. This finding is useful for hypothesis generation, but cartographic comparisons, such as lag-correlation analysis, cannot establish causality. Further spatial analyses should account for age structure, migration, urbanization, access to health care, socioeconomic context, intensity of agricultural production, and the effects of the pandemic and wartime disruptions on both population structure and case registration.

This study has several important limitations. First, the ecological design precludes individual-level inference, and an ecological fallacy cannot be excluded, since national pesticide use indicators do not capture personal exposure, route-specific dose, mixture composition, or protective practices. Second, the available time series were short, especially in the pre-COVID analytical window, which made lag-specific estimates highly sensitive to single observations and to the underlying trend structure. Third, multiple comparisons across many pesticide indicators and lags increase the probability of chance findings, even when this was addressed using BH-FDR correction. Residual false-positive findings therefore remain possible despite statistical correction procedures. Fourth, major confounding factors, including smoking, alcohol use, diet, obesity, screening coverage, ambient air pollution, migration, and regional differences in health-care access, were not directly controlled for. Finally, total pesticide use indicators do not distinguish between active substances, persistence, toxicological class, or regional patterns of application. For these reasons, the observed associations should be interpreted as preliminary and hypothesis-generating rather than causal.

From a practical perspective, these results suggest that reliable assessment of the pesticide–cancer relationship at the population level requires longer time series; finer exposure characterization; stratification by cancer site, sex, and age; and the incorporation of spatial covariates and analytical models that explicitly address autocorrelation, delayed effects, and confounding. Future studies may additionally benefit from modern computational approaches, including machine learning capable of integrating multidimensional exposure indicators, temporal dynamics, and heterogeneous environmental-health datasets. Such an approach will be necessary to determine whether the lag signal observed here in the 5–11-year window is statistically robust, biologically coherent, and reproducible in independent datasets.

## Conclusion

Pesticide use in Ukraine showed marked temporal variability during 2000–2021, with a peak in 2012, a decline in 2014–2020, and a moderate increase in 2021, while herbicides remained the dominant pesticide group throughout the study period. The incidence of cancer increased from 2014–2019 but was substantially distorted from 2020–2021, most likely because of the impact of the COVID-19 pandemic on case detection and registration.

For the overall cancer incidence indicator, lagged associations with pesticide use measures were highly unstable and largely attenuated after detrending and first-difference analysis, suggesting that much of the aggregate-level signal was driven by shared temporal structure rather than by an autonomous exposure–outcome relationship. In contrast, partly reproducible positive ecological association patterns were observed in the 5–11-year lag window for female breast cancer, female and male colon cancer, and female trachea/bronchus/ lung cancer.

These findings may cautiously align with the delayed and multifactorial nature of carcinogenesis; however, because of the ecological design of the study, ecological fallacy cannot be excluded, and the identified associations should not be interpreted at the individual level. Given the short time series, multiple lag comparisons, possible residual false-positive findings despite BH-FDR correction, and the lack of direct control for major confounding factors, the observed associations should be regarded as hypothesis-generating rather than causal. Further research should rely on longer time series, more detailed exposure characterization, stratification by cancer site, sex, and age, and analytical models that account for temporal structure, delayed effects, and confounding. Future studies may also benefit from spatially resolved exposure data and advanced computational approaches for environmental-health modeling.

## Data Availability

The original contributions presented in the study are included in the article/Supplementary material, further inquiries can be directed to the corresponding author.
