# Supplementary material for: An ecological time series study of lagged associations between pesticide use and cancer incidence in Ukraine
Source: Front Public Health. 2026 Jul 1;14:1860025. doi: 10.3389/fpubh.2026.1860025 (PMC13368739; doi:10.3389/fpubh.2026.1860025)
Supplement: Supplementary file 1 [file Table_1.DOCX]

Supplementary Table S1. Spearman lag correlations between pesticide-use indicators and cancer incidence in Ukraine for shifted exposure windows, with Benjamini-Hochberg FDR correction (2014-2021 and 2014-2019 scenarios)

| **Spearman's rank correlation (2014-2021) with BH-FDR** | | | | | | | | | | | | | | | |
| --- | --- | --- | --- | --- | --- | --- | --- | --- | --- | --- | --- | --- | --- | --- | --- |
| **Variables** | Lag-14 | Lag-13 | Lag-12 | Lag-11 | Lag-10 | Lag-9 | Lag-8 | Lag-7 | Lag-6 | Lag-5 | Lag-4 | Lag-3 | Lag-2 | Lag-1 | Lag-0 |
| State Statistics, kg/ha | 0,02 | 0,50 | 0,86 | 0,69 | 0,69 | 0,67 | 0,74 | 0,88 | 0,81 | 0,48 | -0,24 | -0,69 | -0,76 | -0,86 | -0,95** |
| FAOSTAT, kg/ha | 0,02 | 0,50 | 0,83 | 0,72 | 0,72 | 0,71 | 0,78 | 0,91* | 0,81 | 0,48 | -0,24 | -0,69 | -0,76 | -0,86 | -0,95** |
| Fungicides and Bactericides | -0,26 | 0,38 | 0,79 | 0,74 | 0,74 | 0,83 | 0,76 | 0,88 | 0,74 | 0,24 | -0,43 | -0,74 | -0,83 | -0,81 | -0,91* |
| Herbicides | 0,02 | 0,50 | 0,86 | 0,69 | 0,69 | 0,67 | 0,74 | 0,88 | 0,81 | 0,48 | -0,24 | -0,69 | -0,76 | -0,86 | -0,95** |
| Insecticides | -0,26 | 0,38 | 0,79 | 0,74 | 0,74 | 0,74 | 0,71 | 0,83 | 0,95* | 0,64 | -0,02 | -0,50 | -0,79 | -0,74 | -0,83 |
| Other Pesticides | 0,52 | 0,75 | 0,71 | 0,73 | 0,74 | 0,81 | 0,71 | 0,81 | 0,88 | 0,83 | 0,33 | -0,31 | -0,59 | -0,86 | -0,95** |
| Plant Growth Regulators | -0,08 | 0,31 | 0,52 | 0,65 | 0,73 | 0,76 | 0,86 | 0,95* | 0,74 | 0,41 | 0,07 | -0,31 | -0,41 | 0,33 | 0,64 |
| Pesticides (total) | 0,02 | 0,50 | 0,79 | 0,74 | 0,74 | 0,74 | 0,81 | 0,93* | 0,81 | 0,48 | -0,24 | -0,69 | -0,76 | -0,86 | -0,95** |
| Notes: «Lag-14» – 2000-2007 / 2014-2021; «Lag-13» – 2001-2008 / 2014-2021; «Lag-12» – 2002-2009 / 2014-2021; «Lag-11» – 2003-2010 / 2014-2021; «Lag-10» – 2004-2011 / 2014-2021; «Lag-9» – 2005-2012 / 2014-2021; «Lag-8» – 2006-2013 / 2014-2021; «Lag-7» – 2007-2014 / 2014-2021; «Lag-6» – 2008-2015 / 2014-2021; «Lag-5» – 2009-2016 / 2014-2021; «Lag-4» – 2010-2017 / 2014-2021; «Lag-3» – 2011-2018 / 2014-2021; «Lag-2» – 2012-2019 / 2014–-2021; «Lag-1» – 2013-2020 / 2014-2021; «Lag-0» – 2014-2021 / 2014-2021. | | | | | | | | | | | | | | | |
| **Spearman's rank correlation (2014-2019) with BH-FDR** | | | | | | | | | | | | | | | |
| **Variables** | Lag-14 | Lag-13 | Lag-12 | Lag-11 | Lag-10 | Lag-9 | Lag-8 | Lag-7 | Lag-6 | Lag-5 | Lag-4 | Lag-3 | Lag-2 | Lag-1 | Lag-0 |
| State Statistics, kg/ha | -0,37 | 0,49 | 0,83 | 1,00*** | 0,83 | 0,83 | 0,83 | 0,83 | 0,89* | 0,60 | 0,03 | -0,83 | -1,00*** | -1,00*** | -1,00*** |
| FAOSTAT, kg/ha | -0,37 | 0,49 | 0,83 | 1,00*** | 0,90* | 0,90* | 0,90* | 0,90* | 0,89* | 0,60 | 0,03 | -0,83 | -1,00*** | -1,00*** | -1,00*** |
| Fungicides and Bactericides | -0,60 | 0,26 | 0,83 | 1,00*** | 0,94* | 0,94* | 0,94* | 0,89* | 0,77 | 0,49 | -0,37 | -0,94 | -0,94* | -1,00*** | -0,94** |
| Herbicides | -0,37 | 0,49 | 0,83 | 1,00*** | 0,83 | 0,83 | 0,83 | 0,83 | 0,89* | 0,60 | 0,03 | -0,83 | -1,00*** | -1,00*** | -1,00*** |
| Insecticides | -0,60 | 0,26 | 0,83 | 1,00*** | 0,94* | 0,94* | 0,94* | 0,94* | 0,94* | 0,94 | 0,37 | -0,49 | -0,83 | -1,00*** | -0,94** |
| Plant Growth Regulators | - | - | 0,66 | 0,84 | 0,94* | 0,99** | 1,00*** | 1,00*** | 0,94* | 0,60 | 0,09 | -0,31 | -0,43 | 0,14 | 1,00*** |
| Other Pesticides | 0,66 | 0,84 | 0,94 | 0,93* | 0,94* | 0,94* | 0,94* | 0,89* | 0,94* | 0,89 | 0,71 | -0,14 | -0,66 | -1,00*** | -1,00*** |
| Pesticides (total) | -0,37 | 0,49 | 0,83 | 1,00*** | 0,94* | 0,94* | 0,94* | 0,94* | 0,89* | 0,60 | 0,03 | -0,83 | -1,00*** | -1,00*** | -1,00*** |
| Notes: «Lag-14» – 2000-2005 / 2014-2019; «Lag-13» – 2001-2006 / 2014-2019; «Lag-12» – 2002-2007 / 2014-2019; «Lag-11» – 2003-2008 / 2014-2019; «Lag-10» – 2004-2009 / 2014-2019; «Lag-9» – 2005-2010 / 2014-2019; «Lag-8» – 2006-2011 / 2014-2019; «Lag-7» – 2007-2012 / 2014-2019; «Lag-6» – 2008-2013 / 2014-2019; «Lag-5» – 2009-2014 / 2014-2019; «Lag-4» – 2010-2015 / 2014-2019; «Lag-3» – 2011-2016 / 2014-2019; «Lag-2» – 2012-2017 / 2014-2019; «Lag-1» – 2013-2018 / 2014-2019; «Lag-0» – 2014-2019 / 2014-2019. | | | | | | | | | | | | | | | |

Supplementary Table S2. Spearman lag correlations between pesticide-use indicators and breast cancer incidence in women and men, Ukraine, 2014-2019, with Benjamini-Hochberg FDR correction.

| **Women** | | | | | | | | | | | | | | | |
| --- | --- | --- | --- | --- | --- | --- | --- | --- | --- | --- | --- | --- | --- | --- | --- |
| **Variables** | Lag-14 | Lag-13 | Lag-12 | Lag-11 | Lag-10 | Lag-9 | Lag-8 | Lag-7 | Lag-6 | Lag-5 | Lag-4 | Lag-3 | Lag-2 | Lag-1 | Lag-0 |
| State Statistics, kg/ha | -0,54 | 0,43 | 0,77 | 0,94 | 0,94* | 0,66 | 0,77 | 0,77 | 0,94 | 0,71 | 0,09 | -0,77 | -0,94 | -0,94* | -0,94* |
| FAOSTAT, kg/ha | -0,56 | 0,35 | 0,84 | 0,94 | 0,99** | 0,75 | 0,84 | 0,84 | 0,94 | 0,71 | -0,03 | -0,77 | -0,94 | -0,94* | -0,94* |
| Fungicides and Bactericides | -0,71 | 0,14 | 0,77 | 0,94 | 1,00*** | 0,83 | 0,89 | 0,94* | 0,71 | 0,60 | -0,26 | -0,89 | -0,89 | -0,94* | -1,00*** |
| Herbicides | -0,54 | 0,43 | 0,77 | 0,94 | 0,94* | 0,66 | 0,77 | 0,77 | 0,94 | 0,71 | 0,09 | -0,77 | -0,94 | -0,94* | -0,94* |
| Insecticides | -0,71 | 0,14 | 0,77 | 0,94 | 1,00*** | 0,83 | 0,89 | 0,89 | 0,89 | 1,00*** | 0,54 | -0,43 | -0,77 | -0,94* | -1,00*** |
| Other Pesticides | - | - | 0,39 | 0,78 | 0,88 | 0,93 | 0,94* | 0,94* | 1,00*** | 0,71 | 0,03 | -0,43 | -0,49 | 0,09 | 0,94* |
| Plant Growth Regulators | 0,39 | 0,78 | 0,88 | 0,99* | 0,83 | 0,89 | 0,89 | 0,94* | 0,83 | 0,94 | 0,77 | 0,03 | -0,60 | -0,94* | -0,94* |
| Pesticides (total) | -0,54 | 0,43 | 0,77 | 0,94 | 1,00*** | 0,83 | 0,89 | 0,89 | 0,94 | 0,71 | 0,09 | -0,77 | -0,94 | -0,94* | -0,94* |
| Notes: «Lag-14» – 2000-2007 / 2014-2021; «Lag-13» – 2001-2008 / 2014-2021; «Lag-12» – 2002-2009 / 2014-2021; «Lag-11» – 2003-2010 / 2014-2021; «Lag-10» – 2004-2011 / 2014-2021; «Lag-9» – 2005-2012 / 2014-2021; «Lag-8» – 2006-2013 / 2014-2021; «Lag-7» – 2007-2014 / 2014-2021; «Lag-6» – 2008-2015 / 2014-2021; «Lag-5» – 2009-2016 / 2014-2021; «Lag-4» – 2010-2017 / 2014-2021; «Lag-3» – 2011-2018 / 2014-2021; «Lag-2» – 2012-2019 / 2014–-2021; «Lag-1» – 2013-2020 / 2014-2021; «Lag-0» – 2014-2021 / 2014-2021. | | | | | | | | | | | | | | | |
| **Men** | | | | | | | | | | | | | | | |
| **Variables** | Lag-14 | Lag-13 | Lag-12 | Lag-11 | Lag-10 | Lag-9 | Lag-8 | Lag-7 | Lag-6 | Lag-5 | Lag-4 | Lag-3 | Lag-2 | Lag-1 | Lag-0 |
| State Statistics, kg/ha | -0,33 | 0,27 | 0,21 | 0,58 | 0,58 | 0,21 | 0,82 | 0,40 | 0,52 | 0,33 | 0,21 | -0,21 | -0,58 | -0,58 | -0,58 |
| FAOSTAT, kg/ha | -0,28 | 0,06 | 0,31 | 0,58 | 0,65 | 0,28 | 0,77 | 0,49 | 0,52 | 0,33 | 0,12 | -0,21 | -0,58 | -0,58 | -0,58 |
| Fungicides and Bactericides | -0,33 | -0,03 | 0,21 | 0,58 | 0,70 | 0,33 | 0,70 | 0,70 | 0,03 | 0,58 | 0,03 | -0,58 | -0,40 | -0,58 | -0,70 |
| Herbicides | -0,33 | 0,27 | 0,21 | 0,58 | 0,58 | 0,21 | 0,82 | 0,40 | 0,52 | 0,33 | 0,21 | -0,21 | -0,58 | -0,58 | -0,58 |
| Insecticides | -0,33 | -0,03 | 0,21 | 0,58 | 0,70 | 0,33 | 0,70 | 0,58 | 0,40 | 0,70 | 0,33 | -0,27 | -0,21 | -0,58 | -0,70 |
| Plant Growth Regulators | 0,14 | 0,57 | 0,42 | 0,62 | 0,33 | 0,70 | 0,58 | 0,52 | 0,33 | 0,82 | 0,33 | -0,03 | -0,15 | -0,58 | -0,58 |
| Other Pesticides | - | - | 0,14 | 0,57 | 0,42 | 0,49 | 0,58 | 0,58 | 0,70 | 0,33 | 0,09 | 0,21 | -0,33 | -0,33 | 0,58 |
| Pesticides (total) | -0,33 | 0,27 | 0,21 | 0,58 | 0,70 | 0,33 | 0,70 | 0,58 | 0,52 | 0,33 | 0,21 | -0,21 | -0,58 | -0,58 | -0,58 |
| Notes: «Lag-14» – 2000-2005 / 2014-2019; «Lag-13» – 2001-2006 / 2014-2019; «Lag-12» – 2002-2007 / 2014-2019; «Lag-11» – 2003-2008 / 2014-2019; «Lag-10» – 2004-2009 / 2014-2019; «Lag-9» – 2005-2010 / 2014-2019; «Lag-8» – 2006-2011 / 2014-2019; «Lag-7» – 2007-2012 / 2014-2019; «Lag-6» – 2008-2013 / 2014-2019; «Lag-5» – 2009-2014 / 2014-2019; «Lag-4» – 2010-2015 / 2014-2019; «Lag-3» – 2011-2016 / 2014-2019; «Lag-2» – 2012-2017 / 2014-2019; «Lag-1» – 2013-2018 / 2014-2019; «Lag-0» – 2014-2019 / 2014-2019. | | | | | | | | | | | | | | | |

Supplementary Table S3. Spearman lag correlations between pesticide-use indicators and colon cancer incidence in women and men, Ukraine, 2014-2019, with Benjamini-Hochberg FDR correction.

| **Women** | | | | | | | | | | | | | | | |
| --- | --- | --- | --- | --- | --- | --- | --- | --- | --- | --- | --- | --- | --- | --- | --- |
| **Variables** | Lag-14 | Lag-13 | Lag-12 | Lag-11 | Lag-10 | Lag-9 | Lag-8 | Lag-7 | Lag-6 | Lag-5 | Lag-4 | Lag-3 | Lag-2 | Lag-1 | Lag-0 |
| State Statistics, kg/ha | -0,37 | 0,43 | 0,71 | 0,89 | 0,71 | 0,83 | 0,60 | 0,89 | 0,66 | 0,54 | 0,03 | -0,71 | -0,89 | -0,89* | -0,89* |
| FAOSTAT, kg/ha | -0,41 | 0,41 | 0,75 | 0,89 | 0,75 | 0,90 | 0,67 | 0,93* | 0,66 | 0,54 | -0,12 | -0,71 | -0,89 | -0,89* | -0,89* |
| Fungicides and Bactericides | -0,54 | 0,26 | 0,71 | 0,89 | 0,77 | 0,94 | 0,71 | 0,83 | 0,77 | 0,20 | -0,14 | -0,94 | -0,77 | -0,89* | -0,77 |
| Herbicides | -0,37 | 0,43 | 0,71 | 0,89 | 0,71 | 0,83 | 0,60 | 0,89 | 0,66 | 0,54 | 0,03 | -0,71 | -0,89 | -0,89* | -0,89* |
| Insecticides | -0,54 | 0,26 | 0,71 | 0,89 | 0,77 | 0,94 | 0,71 | 0,94* | 0,77 | 0,77 | 0,37 | -0,43 | -0,71 | -0,89* | -0,77 |
| Other Pesticides | 0,66 | 0,68 | 0,88 | 0,72 | 0,94* | 0,71 | 0,94* | 0,66 | 0,94 | 0,60 | 0,83 | -0,31 | -0,49 | -0,89* | -0,89* |
| Plant Growth Regulators | - | - | 0,66 | 0,68 | 0,88 | 0,84 | 0,89 | 0,89 | 0,77 | 0,54 | 0,14 | -0,31 | -0,26 | 0,03 | 0,89* |
| Pesticides (total) | -0,37 | 0,43 | 0,71 | 0,89 | 0,77 | 0,94 | 0,71 | 0,94* | 0,66 | 0,54 | 0,03 | -0,71 | -0,89 | -0,89* | -0,89* |
| Notes: «Lag-14» – 2000-2007 / 2014-2021; «Lag-13» – 2001-2008 / 2014-2021; «Lag-12» – 2002-2009 / 2014-2021; «Lag-11» – 2003-2010 / 2014-2021; «Lag-10» – 2004-2011 / 2014-2021; «Lag-9» – 2005-2012 / 2014-2021; «Lag-8» – 2006-2013 / 2014-2021; «Lag-7» – 2007-2014 / 2014-2021; «Lag-6» – 2008-2015 / 2014-2021; «Lag-5» – 2009-2016 / 2014-2021; «Lag-4» – 2010-2017 / 2014-2021; «Lag-3» – 2011-2018 / 2014-2021; «Lag-2» – 2012-2019 / 2014–-2021; «Lag-1» – 2013-2020 / 2014-2021; «Lag-0» – 2014-2021 / 2014-2021. | | | | | | | | | | | | | | | |
| **Men** | | | | | | | | | | | | | | | |
| **Variables** | Lag-14 | Lag-13 | Lag-12 | Lag-11 | Lag-10 | Lag-9 | Lag-8 | Lag-7 | Lag-6 | Lag-5 | Lag-4 | Lag-3 | Lag-2 | Lag-1 | Lag-0 |
| State Statistics, kg/ha | -0,23 | 0,70 | 0,81 | 0,90 | 0,72 | 0,64 | 0,70 | 0,99** | 0,75 | 0,41 | -0,23 | -0,81 | -0,90 | -0,90* | -0,90* |
| FAOSTAT, kg/ha | -0,22 | 0,64 | 0,82 | 0,90 | 0,79 | 0,75 | 0,73 | 1,00*** | 0,75 | 0,41 | -0,37 | -0,81 | -0,90 | -0,90* | -0,90* |
| Fungicides and Bactericides | -0,41 | 0,49 | 0,81 | 0,90 | 0,84 | 0,84 | 0,75 | 0,93* | 0,64 | 0,12 | -0,32 | -0,99* | -0,81 | -0,90* | -0,84 |
| Herbicides | -0,23 | 0,70 | 0,81 | 0,90 | 0,72 | 0,64 | 0,70 | 0,99** | 0,75 | 0,41 | -0,23 | -0,81 | -0,90 | -0,90* | -0,90* |
| Insecticides | -0,41 | 0,49 | 0,81 | 0,90 | 0,84 | 0,84 | 0,75 | 0,99** | 0,81 | 0,84 | 0,23 | -0,70 | -0,81 | -0,90* | -0,84 |
| Plant Growth Regulators | 0,66 | 0,86 | 0,95 | 0,81 | 0,84 | 0,75 | 0,99* | 0,75 | 0,84 | 0,70 | 0,75 | -0,46 | -0,67 | -0,90* | -0,90* |
| Other Pesticides | - | - | 0,66 | 0,86 | 0,95* | 0,87 | 0,90 | 0,90 | 0,84 | 0,41 | -0,17 | -0,32 | -0,06 | 0,20 | 0,90* |
| Pesticides (total) | -0,23 | 0,70 | 0,81 | 0,90 | 0,84 | 0,84 | 0,75 | 0,99** | 0,75 | 0,41 | -0,23 | -0,81 | -0,90 | -0,90* | -0,90* |
| Notes: «Lag-14» – 2000-2005 / 2014-2019; «Lag-13» – 2001-2006 / 2014-2019; «Lag-12» – 2002-2007 / 2014-2019; «Lag-11» – 2003-2008 / 2014-2019; «Lag-10» – 2004-2009 / 2014-2019; «Lag-9» – 2005-2010 / 2014-2019; «Lag-8» – 2006-2011 / 2014-2019; «Lag-7» – 2007-2012 / 2014-2019; «Lag-6» – 2008-2013 / 2014-2019; «Lag-5» – 2009-2014 / 2014-2019; «Lag-4» – 2010-2015 / 2014-2019; «Lag-3» – 2011-2016 / 2014-2019; «Lag-2» – 2012-2017 / 2014-2019; «Lag-1» – 2013-2018 / 2014-2019; «Lag-0» – 2014-2019 / 2014-2019. | | | | | | | | | | | | | | | |

Supplementary Table S4. Spearman lag correlations between pesticide-use indicators and trachea, bronchus, and lung cancer incidence in women and men, Ukraine, 2014-2019, with Benjamini-Hochberg FDR correction.

| **Women** | | | | | | | | | | | | | | | |
| --- | --- | --- | --- | --- | --- | --- | --- | --- | --- | --- | --- | --- | --- | --- | --- |
| **Variables** | Lag-14 | Lag-13 | Lag-12 | Lag-11 | Lag-10 | Lag-9 | Lag-8 | Lag-7 | Lag-6 | Lag-5 | Lag-4 | Lag-3 | Lag-2 | Lag-1 | Lag-0 |
| State Statistics, kg/ha | -0,49 | 0,41 | 0,72 | 0,93 | 0,90 | 0,64 | 0,84 | 0,70 | 0,93 | 0,67 | 0,12 | -0,72 | -0,93 | -0,93* | -0,93* |
| FAOSTAT, kg/ha | -0,52 | 0,31 | 0,81 | 0,93 | 0,96* | 0,72 | 0,90 | 0,78 | 0,93 | 0,67 | 0,00 | -0,72 | -0,93 | -0,93* | -0,93* |
| Fungicides and Bactericides | -0,67 | 0,12 | 0,72 | 0,93 | 0,99** | 0,78 | 0,93 | 0,90 | 0,64 | 0,67 | -0,29 | -0,84 | -0,87 | -0,93* | -0,99* |
| Herbicides | -0,49 | 0,41 | 0,72 | 0,93 | 0,90 | 0,64 | 0,84 | 0,70 | 0,93 | 0,67 | 0,12 | -0,72 | -0,93 | -0,93* | -0,93* |
| Insecticides | -0,67 | 0,12 | 0,72 | 0,93 | 0,99** | 0,78 | 0,93 | 0,84 | 0,87 | 0,99** | 0,49 | -0,41 | -0,72 | -0,93* | -0,99* |
| Other Pesticides | 0,40 | 0,79 | 0,83 | 0,97 | 0,78 | 0,93 | 0,84 | 0,93* | 0,78 | 0,99** | 0,67 | 0,06 | -0,58 | -0,93* | -0,93* |
| Plant Growth Regulators | - | - | 0,40 | 0,79 | 0,83 | 0,91 | 0,93 | 0,93* | 0,99* | 0,67 | 0,06 | -0,32 | -0,52 | 0,06 | 0,93* |
| Pesticides (total) | -0,49 | 0,41 | 0,72 | 0,93 | 0,99** | 0,78 | 0,93 | 0,84 | 0,93 | 0,67 | 0,12 | -0,72 | -0,93 | -0,93* | -0,93* |
| Notes: «Lag-14» – 2000-2007 / 2014-2021; «Lag-13» – 2001-2008 / 2014-2021; «Lag-12» – 2002-2009 / 2014-2021; «Lag-11» – 2003-2010 / 2014-2021; «Lag-10» – 2004-2011 / 2014-2021; «Lag-9» – 2005-2012 / 2014-2021; «Lag-8» – 2006-2013 / 2014-2021; «Lag-7» – 2007-2014 / 2014-2021; «Lag-6» – 2008-2015 / 2014-2021; «Lag-5» – 2009-2016 / 2014-2021; «Lag-4» – 2010-2017 / 2014-2021; «Lag-3» – 2011-2018 / 2014-2021; «Lag-2» – 2012-2019 / 2014–-2021; «Lag-1» – 2013-2020 / 2014-2021; «Lag-0» – 2014-2021 / 2014-2021. | | | | | | | | | | | | | | | |
| **Men** | | | | | | | | | | | | | | | |
| **Variables** | Lag-14 | Lag-13 | Lag-12 | Lag-11 | Lag-10 | Lag-9 | Lag-8 | Lag-7 | Lag-6 | Lag-5 | Lag-4 | Lag-3 | Lag-2 | Lag-1 | Lag-0 |
| State Statistics, kg/ha | 0,46 | -0,12 | -0,35 | -0,67 | -0,70 | -0,41 | -0,81 | -0,29 | -0,70 | -0,55 | -0,35 | 0,35 | 0,67 | 0,67 | 0,67 |
| FAOSTAT, kg/ha | 0,49 | 0,03 | -0,47 | -0,67 | -0,75 | -0,44 | -0,82 | -0,41 | -0,70 | -0,55 | -0,27 | 0,35 | 0,67 | 0,67 | 0,67 |
| Fungicides and Bactericides | 0,55 | 0,17 | -0,35 | -0,67 | -0,78 | -0,46 | -0,81 | -0,64 | -0,29 | -0,84 | 0,12 | 0,52 | 0,58 | 0,67 | 0,78 |
| Herbicides | 0,46 | -0,12 | -0,35 | -0,67 | -0,70 | -0,41 | -0,81 | -0,29 | -0,70 | -0,55 | -0,35 | 0,35 | 0,67 | 0,67 | 0,67 |
| Insecticides | 0,55 | 0,17 | -0,35 | -0,67 | -0,78 | -0,46 | -0,81 | -0,52 | -0,58 | -0,78 | -0,46 | 0,12 | 0,35 | 0,67 | 0,78 |
| Plant Growth Regulators | -0,13 | -0,55 | -0,46 | -0,75 | -0,46 | -0,81 | -0,52 | -0,70 | -0,46 | -0,93 | -0,35 | -0,29 | 0,26 | 0,67 | 0,67 |
| Other Pesticides | - | - | -0,13 | -0,55 | -0,46 | -0,63 | -0,67 | -0,67 | -0,78 | -0,55 | -0,23 | 0,00 | 0,64 | 0,20 | -0,67 |
| Pesticides (total) | 0,46 | -0,12 | -0,35 | -0,67 | -0,78 | -0,46 | -0,81 | -0,52 | -0,70 | -0,55 | -0,35 | 0,35 | 0,67 | 0,67 | 0,67 |
| Notes: «Lag-14» – 2000-2005 / 2014-2019; «Lag-13» – 2001-2006 / 2014-2019; «Lag-12» – 2002-2007 / 2014-2019; «Lag-11» – 2003-2008 / 2014-2019; «Lag-10» – 2004-2009 / 2014-2019; «Lag-9» – 2005-2010 / 2014-2019; «Lag-8» – 2006-2011 / 2014-2019; «Lag-7» – 2007-2012 / 2014-2019; «Lag-6» – 2008-2013 / 2014-2019; «Lag-5» – 2009-2014 / 2014-2019; «Lag-4» – 2010-2015 / 2014-2019; «Lag-3» – 2011-2016 / 2014-2019; «Lag-2» – 2012-2017 / 2014-2019; «Lag-1» – 2013-2018 / 2014-2019; «Lag-0» – 2014-2019 / 2014-2019. | | | | | | | | | | | | | | | |
